# Human cGAS Drives LINE‐1 Transcriptional Activation to Trigger MAVS‐Dependent Cellular Senescence

**DOI:** 10.1111/acel.70484

**Published:** 2026-04-09

**Authors:** Zhixi Chen, Lingjiang Chen, Xinyu Chen, Hao Wang, Huanyin Tang, Zhengyi Zhen, Ying Jiang, Zhiyong Mao, Yu Chen

**Affiliations:** ^1^ Shanghai Key Laboratory of Maternal Fetal Medicine, Clinical and Translational Research Center of Shanghai First Maternity and Infant Hospital, Frontier Science Center for Stem Cell Research, School of Life Sciences and Technology Tongji University Shanghai China

**Keywords:** cellular senescence, cGAS, comparative biology, LINE‐1 retrotransposon, MAVS

## Abstract

Long interspersed nuclear element 1 (LINE‐1 or L1) retrotransposons pose a significant threat to somatic genomic integrity and are a source of sterile inflammation. Consequently, L1 activity is stringently controlled by multiple regulatory layers to ensure silencing, while its transcriptional derepression is linked to aging and age‐related diseases. Recent studies have revealed complex interrelationships between L1 and cGAS, but whether cGAS regulates L1 transcription and its biological significance remains unclear. Here, we demonstrate that human cGAS activates L1 transcription by upregulating the transcriptional regulators CTCF and RUNX3. This cGAS‐mediated promotion of L1 transcription is absent in mice due to functional divergence in CTCF and RUNX3. Furthermore, cGAS‐mediated elevation of L1 mRNA promotes cellular senescence via MAVS, a key RNA‐sensing pathway component. Together, our findings reveal a novel role of cGAS in activating L1 transcription and define a cGAS–L1–MAVS senescence pathway, thereby bridging the noncanonical function of cGAS and the RNA‐sensing signaling.

## Graphical abstract

cGAS activates L1 retrotransposon transcription by upregulating CTCF and RUNX3. Elevated L1 mRNA triggers cellular senescence via MAVS, a key adaptor in RNA‐sensing pathways. Notably, mouse CTCF/RUNX3 could not activate L1 transcription due to functional divergence. Our findings define a human‐specific cGAS–L1–MAVS senescence axis, revealing the noncanonical pro‐senescent role of cGAS.

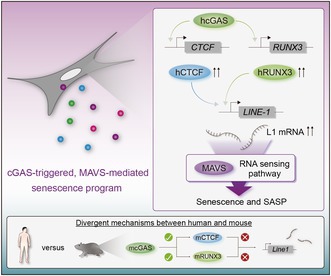

## 1 Introduction

Long interspersed nuclear element 1 (LINE‐1, L1) retrotransposons constitute ~17% of the human genome and represent the only active autonomous transposable elements in humans. While the majority of L1 copies are transcriptionally silent, several retain the capacity to transcribe mRNA, which is translated into functional proteins ORF1p and ORF2p. These proteins assemble with L1 mRNA to form a ribonucleoprotein complex that propagates across the host genome via target‐primed reverse transcription (Ghanim et al. 2025). L1 plays diverse functional roles throughout life stages, from embryonic development to biological aging (De Cecco et al. 2019; Jachowicz et al. 2017; Li, Yu, et al. 2024; Simon et al. 2019). However, L1 retrotransposition induces DNA damage and is considered mutagenic (Gasior et al. 2006; Simon et al. 2019). Consequently, L1 transcription is tightly restricted through multiple layers, including DNA and histone modifications, RNA processing, transcription factors, and posttranslational control of protein localization, activity, and degradation (Li and Liu 2025), and its derepression is associated with aging and multiple diseases.

Cyclic GMP‐AMP synthase (cGAS) is a primary cytosolic DNA sensor that recognizes endogenous and exogenous DNA to initiate innate immune responses (Sun et al. 2013). Recent studies implicate cGAS in regulating cellular senescence (Dou et al. 2017; Gluck et al. 2017; Yang et al. 2017), and it can also translocate into the nucleus to modulate DNA repair, telomere homeostasis, and replication fork stability (Chen, Chen, et al. 2020; Jiang et al. 2019; Li et al. 2022; Liu et al. 2018; Zhang et al. 2024). Additionally, our previous work demonstrated that cGAS facilitates L1 ORF2p degradation to maintain genomic stability (Zhen et al. 2023). Although the reverse transcription and retrotransposition of L1 is stringently controlled and generally rare (Freeman et al. 2011; Kano et al. 2009), active transcription occurs under specific conditions and can contribute to detrimental processes like aging (De Cecco et al. 2019; Simon et al. 2019). Whether and how cGAS regulates L1 transcription and the resulting biological consequences are still unclear.

Chronic inflammation is closely linked to aging (Chen, Geng, et al. 2020; Kroemer et al. 2025), with endogenous nucleic acids proposed as key drivers of inflammaging, influencing aging rate and disease susceptibility (Franceschi et al. 2018). Cellular senescence, a state of irreversible cell cycle arrest, is characterized by increased synthesis and secretion of extracellular modulators, including proteins, lipids, and extracellular vesicles—termed senescence‐associated secretory phenotype (SASP) (Wang et al. 2024). Many SASP factors are pro‐inflammatory. Accumulating evidence shows that cGAS‐mediated DNA sensing initiates SASP factor production (Gluck et al. 2017; Victorelli et al. 2023; Yang et al. 2017), which reinforces senescence via an autocrine manner or propagates it via a paracrine manner (Wang et al. 2024), and contributes to chronic inflammation. Beyond DNA, RNA species can activate the intracellular RNA‐sensing pathway via mitochondrial antiviral‐signaling protein (MAVS), triggering NF‐κB‐ and IRF‐mediated synthesis of inflammatory cytokines and interferons (Li, Zhu, et al. 2024). Although DNA fragments originating from nuclear or mitochondrial damage, along with L1 cDNA, are well‐known inducers of SASP and inflammation (Chen et al. 2024, 2023; De Cecco et al. 2019; Gulen et al. 2023; Victorelli et al. 2023; Yang et al. 2017; Zhang et al. 2022), the roles of RNA fragments and the RNA‐sensing pathway in SASP and senescence establishment remain unexplored.

In this study, we demonstrate that human cGAS, contrary to its role in promoting L1 ORF2p degradation (Zhen et al. 2023), facilitates L1 mRNA transcription. Mechanistically, cGAS localizes to promoter regions and upregulates two positive transcriptional regulators of L1, CTCF and RUNX3. Notably, this activation is human specific, as mouse homologs of CTCF and RUNX3 lack this regulatory capacity. In addition, human cGAS promotes stress‐induced premature senescence and SASP factor secretion. Critically, depletion of MAVS, a core component of the RNA‐sensing pathway, or of L1 abolishes these pro‐senescence effects mediated by cGAS. Taken together, our findings reveal a noncanonical, species‐divergent function of cGAS and establish a functional nexus between cGAS and the MAVS‐mediated RNA‐sensing pathway in driving cellular senescence.

## 2 Materials and Methods

### 2.1 Cell Culture and Transfection

HCA2‐hTERT, HeLa, mouse skin fibroblasts (MSFs), HEK‐293FT cells, and their derived cell lines were maintained in Dulbecco's Modified Eagle's Medium (Corning, Cat. #10‐013‐CVR) supplemented with 10% fetal bovine serum (Gibco, Cat. #10270‐106), 1% nonessential amino acids (Gibco, Cat. #11140‐050), and 1% penicillin–streptomycin (Gibco, Cat. #15140‐122). All cells were cultured at 37°C in a humidified incubator (Thermo Scientific HERAcell 240i) with 5% CO_2_ and were routinely tested for mycoplasma contamination using PCR. HCA2‐hTERT and MSFs were transfected using the Lonza 4D Nucleofector system with program DT‐130, while HeLa cells were transfected using program CN‐114. HEK‐293FT cells were transfected via polyethylenimine (PEI)‐mediated method.

### 2.2 Plasmids and Reagents

The full‐length coding sequences (CDS) of human cGAS, CTCF, and RUNX3 were amplified by PCR using HeLa cDNA as template. The CDS of mouse cGAS, CTCF, and RUNX3 was amplified using MSF cDNA as template. All amplified CDS were cloned into HA‐tag or Flag‐tag vector and validated by Sanger DNA sequencing. shRNA sequences were listed in Table S1. The following primary antibodies were used: anti‐cGAS (ABclonal, Cat. #A8335), anti‐HA (Cell Signaling Technology, Cat. #3724), anti‐Flag (ABclonal, Cat. #AE005), anti‐p21 (ABclonal, Cat. #A19094), anti‐MAVS (ABclonal, Cat. # A25005), and anti‐GAPDH (Proteintech, Cat. #60004). FITC‐Phalloidin (Cat. #RM02836) was purchased from ABclonal.

### 2.3 Analysis of the L1‐5′ UTR Promoter Activity

HeLa cells were co‐transfected with the L1‐5′ UTR‐firefly luciferase reporter (Athanikar et al. 2004; Van Meter et al. 2014) and a vector encoding Renilla luciferase as an internal control. Twenty‐four hours post‐transfection, cells were harvested and lysed. Luciferase activity was measured using a GloMax Luminometer (Promega, Cat. #E5311). Relative L1‐5′ UTR promoter activity was calculated as the ratio of Firefly luciferase luminescence to Renilla luciferase luminescence.

### 2.4 RNA Extraction and Quantitative Real‐Time PCR

Cells were lysed and total RNA was extracted using the RNASimple Total RNA kit (Tiangen, Cat. #DP419) 24 h post‐transfection. Subsequently, 1 μg of RNA was reverse‐transcribed into cDNA using the TransScript II Reverse Transcriptase kit (Trans, Cat. #AT311‐02). The resulting cDNA was used as the template for quantitative real‐time PCR using SYBR Green qPCR Mix (Roche, Cat. #04913914001) on a ViiA 7 Real‐Time PCR System (Applied Biosystems). RT‐qPCR was performed using three independent biological replicates, yielding consistent findings. Data from one representative replicate are shown in the figures. Primer sequences are listed in Table S2. The primer sequences designed to preferentially amplify elements of the full‐length human‐specific L1HS subfamily were previously reported (De Cecco et al. 2019).

### 2.5 Chromatin Immunoprecipitation (ChIP)

ChIP assays were conducted as previously described (Chen et al. 2023; Jiang et al. 2025). Briefly, to assess cGAS enrichment on the CTCF or RUNX3 promoters, HEK‐293FT cells were transfected with empty vector or HA‐tagged cGAS vector. Cells were harvested 24 h post‐transfection for ChIP analysis using HA‐specific antibodies or control IgG. Precipitated DNA from anti‐HA and IgG samples, along with input samples, was quantified by real‐time PCR using the 2^−∆∆CT^ method. Relative enrichment was calculated as the ratio of immunoprecipitated DNA to input chromatin, with the value obtained for the IgG sample or empty vector‐transfected sample set to 1. Primer sequences for CTCF and RUNX3 promoters are listed in Table S2.

### 2.6 Annotation of cGAS ChIP‐Seq Peaks

cGAS ChIP‐seq data were previously reported in Gentili et al. (2019). Filtered human cGAS peaks from the GSE125475 dataset were annotated using the annotatePeaks.pl. script from HOMER software (version 4.11). The full‐length L1 annotation file was obtained from the L1base2 database.

### 2.7 Analysis of cGAS Binding Pattern on L1

cGAS ChIP‐seq data were previously reported in Gentili et al. (2019) and are available under accession number GSE125431. Reads were trimmed with fastp 0.23.4, mapped to GRCh38 with Bowtie2 2.5.4 (very‐sensitive, *k* = 100), and multimappers were reallocated by Allo 1.2.0. Duplicates were marked with samblaster 0.1.26 on name‐sorted BAMs, then coordinate‐sorted and indexed with sambamba 1.0.1. Replicates were merged per condition. Genome‐wide log_2_(cGAS/NLS) tracks were generated with deepTools bamCompare using SES scaling, and signal over L1 elements was extracted with computeMatrix and visualized with plotProfile.

### 2.8 Correlation Analysis of cGAS Expression With CTCF and RUNX3

Using GTEx (V8) data, TPM values were log‐transformed as ln (TPM + 0.00001). Pearson correlation analysis between *CGAS* and *CTCF*/*RUNX3* expression was performed using R (version 4.2.2), with visualizations generated using the ggplot2 package (version 3.5.1).

### 2.9 Senescence‐Associated β‐Galactosidase (SA‐β‐Gal) Staining

Cells were irradiated with 6 Gy X‐ray at 24 h post‐transfection. Six days postirradiation, cells were harvested, washed with PBS, and fixed for 5 min at room temperature using a solution containing 2% formaldehyde and 0.2% glutaraldehyde. After three PBS washes, cells were incubated overnight in staining solution (1 mg/mL X‐gal, 40 mM citric acid/sodium phosphate buffer (pH 6.0), 5 mM potassium ferrocyanide, 5 mM potassium ferricyanide, 150 mM NaCl, 2 mM MgCl_2_) at 37°C. The next day, stained cells were imaged using an inverted microscope and quantified in a blinded manner.

### 2.10 Quantification of Cell Surface Area

Cells were washed once with PBS, fixed with 4% paraformaldehyde in PBS for 20 min at room temperature, and then washed twice with PBS. Permeabilization was performed using 0.3% Triton X‐100 in PBS for 10 min at room temperature, followed by two PBS washes. Cells were then stained with FITC‐phalloidin working solution (1:200 dilution) for 20 min at room temperature in the dark and washed twice with PBS. Finally, cells were mounted using a DAPI‐containing mounting medium.

### 2.11 Statistical Analysis

No statistical methods were used to predetermine sample size. Sample sizes were determined based on experience with similar experiments. Exact *p* values are reported in the figures. Analysis was performed using GraphPad Prism (version 10) software.

## 3 Results

### 3.1 Human cGAS Transcriptionally Activates L1

**FIGURE 1:**
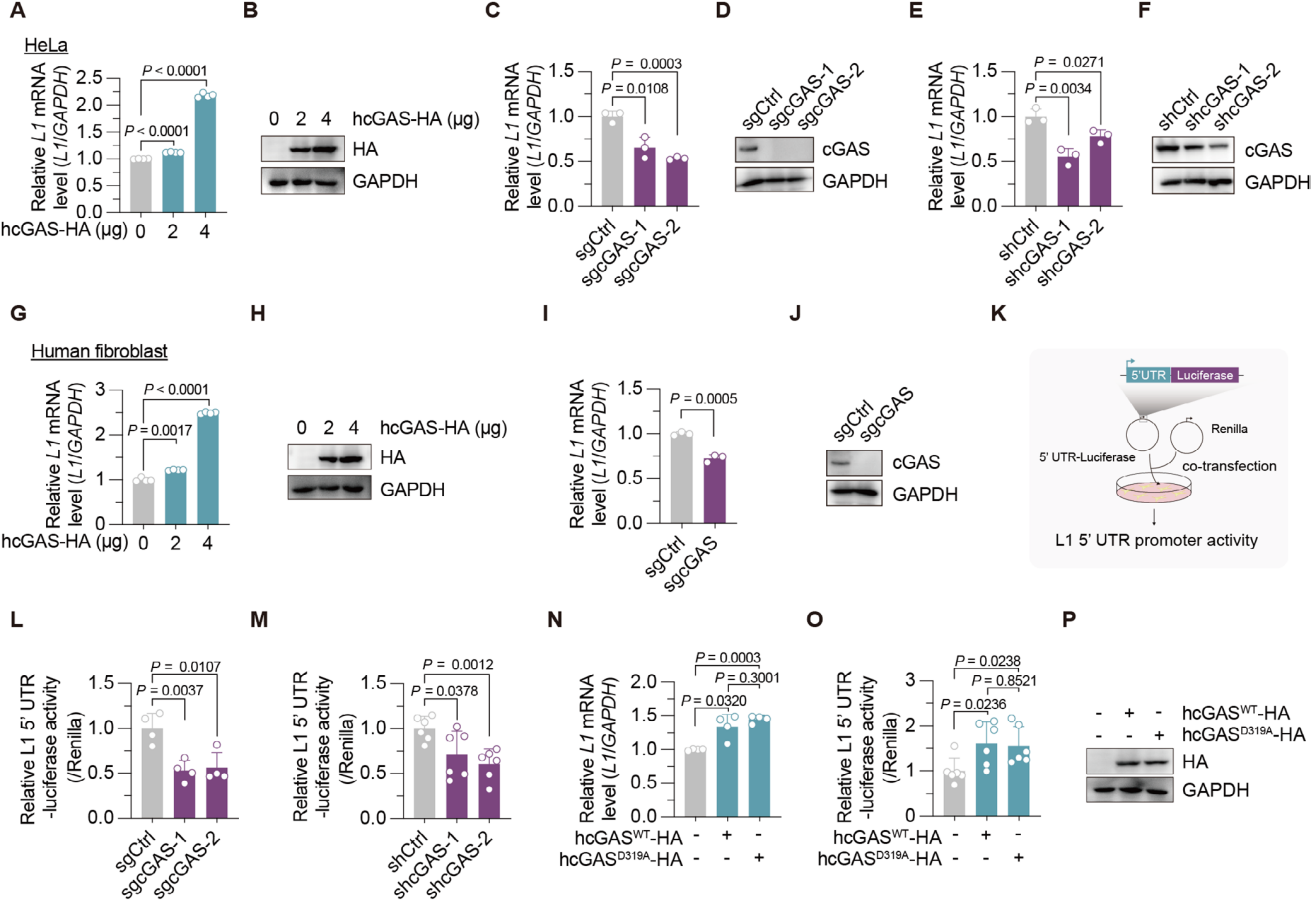
Human cGAS promotes L1 transcription. (A, B) The effect of human cGAS overexpression on *L1* mRNA level in HeLa cells. Cells were transfected with empty vector or vectors encoding human cGAS, and RNA was extracted at 24 h post‐transfection. Empty vector was co‐transfected to ensure the total amount of vectors transfected was equal. Overexpression was confirmed by Western blotting. (C, D) The effect of CRISPR/Cas9‐mediated cGAS depletion on *L1* mRNA levels in HeLa cells. Western blotting confirms endogenous cGAS expression. (E, F) The effect of shRNA‐mediated cGAS knockdown on *L1* mRNA levels in HeLa cells. Western blotting confirms endogenous cGAS expression. (G, H) The mRNA level of *L1* in human skin fibroblast HCA2‐hTERT cells transfected with vectors encoding human cGAS. (I, J) The *L1* mRNA level in cGAS‐depleted HCA2‐hTERT cells. Western blotting confirms endogenous cGAS expression. (K) Schematic illustration of the experiment analyzing L1 5′ UTR promoter activity. Cells were co‐transfected with a Firefly luciferase reporter vector containing the L1 5′ UTR promoter sequence and a Renilla luciferase plasmid serving as an internal control. (L) L1 5′ UTR promoter activity in cGAS‐depleted HeLa cells. (M) The effect of cGAS knockdown on L1 5′ UTR promoter activity in HeLa cells. (N) The effect of overexpressing wild‐type human cGAS or its catalytically inactive mutant D319A on *L1* mRNA level in HCA2‐hTERT cells. (O, P) The effect of overexpressing wild‐type human cGAS or mutant D319A on L1 5′ UTR promoter activity in HCA2‐hTERT cells. cGAS protein levels were validated by Western blot. The empty vector was transfected as the negative control. The experiments were repeated for three times.

While our earlier study demonstrated that cGAS promotes degradation of L1 ORF2p at the protein level, its role in L1 transcription was unknown. RT‐qPCR analysis in HeLa cells transfected with human cGAS revealed a dose‐dependent increase in L1 mRNA levels (Figure 1A,B). This effect was confirmed using alternative primer pairs (Figure S1). Conversely, CRISPR/Cas9‐mediated depletion of *CGAS* significantly reduced L1 mRNA (Figure 1C,D), and shRNA‐mediated gene silencing yielded similar results (Figure 1E,F). We also confirmed the effects of cGAS on L1 mRNA in immortalized human foreskin fibroblasts, HCA2‐hTERT, where cGAS overexpression elevated, and its depletion suppressed, L1 mRNA levels (Figure 1G–J). To determine if cGAS acts through influencing the L1 promoter activity, we employed an L1 5′ UTR‐Luciferase reporter system (Athanikar et al. 2004; Van Meter et al. 2014; Figure 1K). Both CRISPR/Cas9‐mediated depletion (Figure 1L) and shRNA‐mediated knockdown (Figure 1M) of cGAS significantly repressed L1 5′ UTR promoter activity. Crucially, using the enzymatically inactive cGAS mutant D319A, we found that both wild‐type and mutant cGAS promoted L1 mRNA levels and L1 5′ UTR promoter acitivity (Figure 1N–P). These results indicate that cGAS‐mediated transcriptional activation of L1 is independent of its canonical function.

### 3.2 Human cGAS Regulates L1 in a CTCF‐ and RUNX3‐Dependent Manner

**FIGURE 2:**
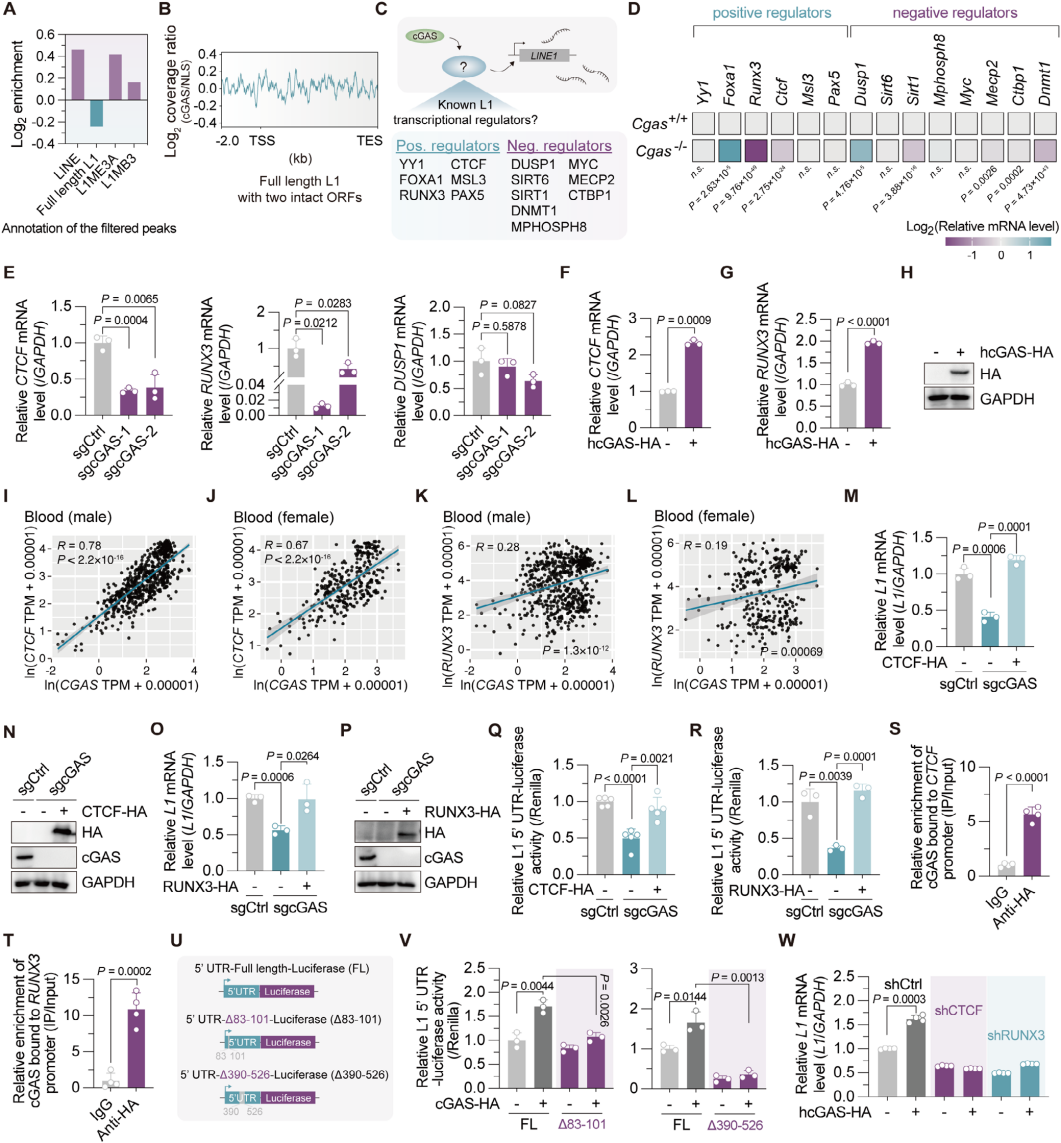
CTCF and RUNX3 mediate human cGAS‐dependent L1 transcriptional activation. (A) Annotation of filtered peaks from previously reported cGAS ChIP‐seq data across LINE‐1 subfamilies. (B) ChIP‐seq analysis of cGAS binding patterns on full‐length retrotranspositionally active L1 DNA. TES, transcription end site; TSS, transcription start site. (C) Previously reported transcriptional regulators of L1. (D) RNA‐seq reanalysis of changes in known L1 regulator expression following *Cgas* depletion in mouse cells. (E) mRNA levels of *CTCF*, *RUNX3*, and *DUSP1* in cGAS‐depleted human cells. (F–H) The effect of human cGAS overexpression on *CTCF* (F) and *RUNX3* (G) mRNA levels. Overexpression was confirmed by Western blotting (H). (I–L) Correlation analysis between *CGAS* expression levels and *CTCF* (I, J) or *RUNX3* (K, L) expression levels in blood using data extracted from GTEx (V8). (M, N) Analysis of *L1* mRNA level in cGAS‐depleted HeLa cells with or without CTCF overexpression. (O, P) Analysis of *L1* mRNA level in cGAS‐depleted HeLa cells with or without RUNX3 overexpression. (Q, R) The effect of CTCF (Q) and RUNX3 (R) overexpression on L1 5′ UTR promoter activity in cGAS‐depleted HeLa cells. (S, T) ChIP‐qPCR analysis of cGAS enrichment at *CTCF* (S) or *RUNX3* (T) promoter. HEK293‐FT cells were transfected with a vector encoding HA‐tagged human cGAS and harvested 24 h post‐transfection for ChIP assay using an anti‐HA antibody. (U, V) The effect of cGAS overexpression on full‐length or truncated L1 5′ UTR promoter activity. (W) The effect of human cGAS overexpression on *L1* mRNA level in HCA2‐hTERT cells stably expressing shRNA targeting CTCF or RUNX3. The empty vector was transfected as the negative control for all the experiments. The experiments were repeated for three times.

Previous study has reported nuclear cGAS enrichment on LINE DNA repeats (Gentili et al. 2019). Interestingly, although not statistically significant, our reanalysis revealed a trend of cGAS preferentially binding to evolutionarily old, retrotranspositionally inactive L1 subfamilies, such as L1ME3A and L1MB3, over retrotranspositionally active full‐length L1 (Abrusan and Krambeck 2006; Figure 2A). When analyzing the cGAS binding pattern on these L1 sequences, we did not observe specific binding of cGAS to the 5′ UTR promoter region of either retrotranspositionally active L1 or the L1ME3A and L1MB3 subfamilies (Figure 2B, Figure S2). These data suggested that the transcriptional regulation of L1 by cGAS is likely indirect. Therefore, we shifted our focus to searching the literature for known transcriptional regulators of L1. We found that a number of both positive regulators (which promote L1 transcription) and negative regulators (which repress L1 transcription) have been reported (Figure 2C). Analysis of a published dataset (Gluck et al. 2017) identified altered expression of several L1 regulators upon cGAS depletion. Among these, the positive regulators RUNX3 and CTCF (Sun et al. 2018; Yang et al. 2003) (downregulated upon *Cgas* depletion), and the negative regulator DUSP1 (Briggs et al. 2021) (upregulated upon *Cgas* depletion), emerged as candidate mediators for cGAS‐induced L1 mRNA changes (Figure 2D). Subsequent RT‐qPCR in cGAS‐depleted HeLa cells confirmed significant reductions in CTCF and RUNX3 mRNA, consistent with RNA‐seq data, while no significant upregulation of DUSP1 was observed (Figure 2E). Consistently, human cGAS overexpression increased the mRNA levels of both CTCF and RUNX3 (Figure 2F–H). In silico analysis of the GTEx dataset further validated positive correlations between cGAS and CTCF or RUNX3 expression across human tissues and sexes (Figure 2I–L, Figure S3), suggesting the impact of cGAS on the expression of CTCF and RUNX3 may be conserved in humans.

Notably, cGAS depletion reduced L1 mRNA levels, and this reduction was rescued by restoring CTCF or RUNX3 expression (Figure 2M–P). Similarly, CTCF or RUNX3 overexpression rescued the diminished L1 5′ UTR promoter activity in cGAS‐depleted cells (Figure 2Q,R). Furthermore, DUSP1 knockdown in cGAS‐depleted cells did not rescue the reductions in L1 mRNA levels or L1 5′ UTR promoter activity (Figure S4A,B). These data demonstrated that DUSP1 is not responsible for cGAS‐mediated activation of L1 transcription. Taken together, our results revealed that cGAS transcriptionally regulates L1 through CTCF and RUNX3. A recent study has reported that cGAS could be recruited to the promoter region to regulate gene expression (Zhang et al. 2024). Supporting this mechanism, our ChIP experiments revealed that cGAS enriched at the promoters of the *CTCF* and *RUNX3* genes, independent of its enzymatic activity (Figure 2S,T, Figure S4C,D), suggesting potential direct regulation of these targets. Due to the lack of commercial antibodies against cGAS suitable for ChIP, we could not confirm endogenous cGAS binding to these promoters. This validation should be addressed in future research. Previous studies (Sun et al. 2018; Yang et al. 2003) and analysis of publicly accessible data further confirmed the enrichment of both CTCF and RUNX3 at the 5′ UTR of the L1 sequence (Figure S4E,F). Moreover, while cGAS significantly enhanced the activity of the full‐length L1 5′ UTR promoter, this effect was markedly diminished upon deletion of either the +83 to +101 region (Yang et al. 2003) or the +390 to +526 region (Sun et al. 2018), which correspond to the binding sites for RUNX3 and CTCF respectively (Figure 2U,V). Additionally, knocking down CTCF or RUNX3 abolished the increase in L1 mRNA induced by cGAS overexpression (Figure 2W), confirming these factors are essential mediators of cGAS‐dependent L1 regulation.

### 3.3 cGAS‐Mediated Regulation of L1 Transcription Is Not Conserved in Mice

**FIGURE 3:**
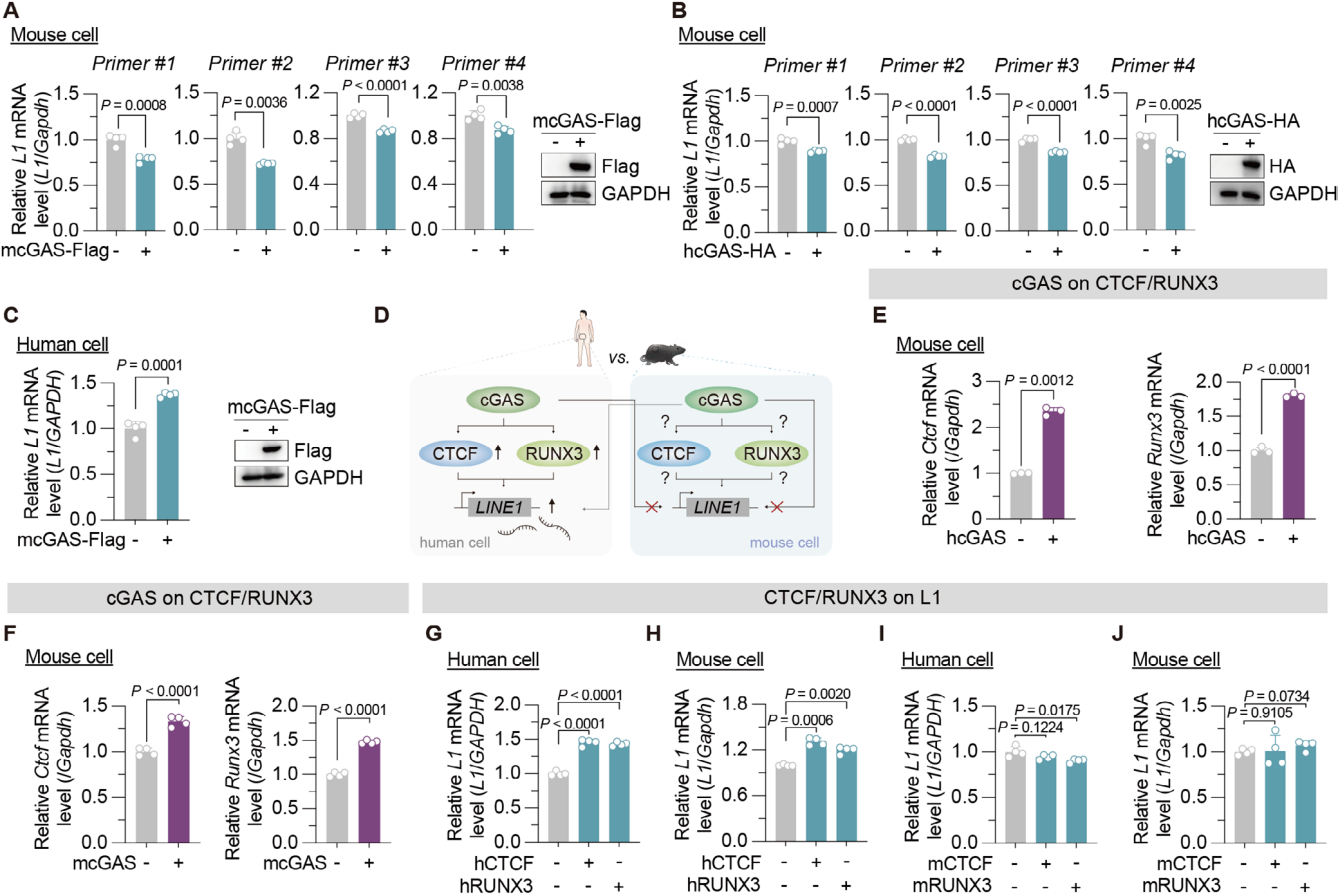
Species‐specific cGAS regulation of L1 transcription through divergent CTCF/RUNX3 function. (A) The effect of mouse cGAS overexpression on *L1* mRNA level in mouse skin fibroblasts (MSFs). (B) The effect of human cGAS overexpression on *L1* mRNA level in MSFs. (C) Analysis of *L1* mRNA level in human HCA2‐hTERT cells with or without mouse cGAS overexpression. (D) Schematic illustration of differences in human or mouse cGAS regulating L1 transcriptional activity. (E) Analysis of *Ctcf* and *Runx3* mRNA levels in MSFs with or without human cGAS overexpression. (F) Analysis of *Ctcf* and *Runx3* mRNA levels in MSFs cells with or without mouse cGAS overexpression. (G) Validation of the effect of human CTCF or RUNX3 overexpression on *L1* mRNA level in human HCA2‐hTERT cells. (H) The effect of human CTCF or RUNX3 overexpression on *L1* mRNA level in MSFs. (I) The effect of mouse CTCF or RUNX3 overexpression on *L1* mRNA level in human HCA2‐hTERT cells. (J) The effect of mouse CTCF or RUNX3 overexpression on *L1* mRNA level in MSFs. The empty vector was transfected as the negative control for all the experiments. The experiments were repeated for three times.

When analyzing the effect of mouse cGAS overexpression on L1 expression in mouse skin fibroblasts, we unexpectedly observed no induction of L1 mRNA; instead, mouse cGAS induced a mild but significant inhibitory effect on L1 transcription. Similar results were obtained using three additional primer pairs (Figure 3A). This discrepancy prompted us to test whether human cGAS could promote L1 expression in mouse cells. However, human cGAS also failed to induce L1 mRNA (Figure 3B). In contrast, mouse cGAS increased L1 mRNA levels in human cells (Figure 3C). These data suggest that the distinct effects of cGAS on L1 expression are independent of functional divergences in cGAS itself, but rather dependent on cellular context (Figure 3D).

We therefore hypothesized that intracellular signaling differences between species are critical determinants. We found that human cGAS overexpression in mouse cells retained the capacity to induce mouse CTCF and RUNX3 expression, mirroring the effect of mouse cGAS (Figure 3E,F). These findings indicate that the critical divergence point likely resides downstream of CTCF/RUNX3 induction, within the CTCF/RUNX3–L1 regulatory axis.

To directly compare the roles of CTCF and RUNX3 across species, we overexpressed human or mouse versions of these factors in both human and mouse cells. Consistent with previous reports, human CTCF and RUNX3 enhanced L1 mRNA levels in human cells (Figure 3G, Figure S5A). Notably, they also promoted L1 transcription in mouse cells (Figure 3H, Figure S5A,B). Conversely, overexpression of mouse CTCF and RUNX3 failed to induce L1 transcription in either human or mouse cells (Figure 3I,J, Figure S5C–E), demonstrating that the regulatory axis linking CTCF/RUNX3 to L1 expression is not conserved.

Why is this regulatory axis not conserved? One possibility is evolutionary divergence, where sequence variability—particularly in the CTCF/RUNX3 amino acid residues—affects their binding affinity to L1. Protein sequence alignment revealed that while the DNA‐binding domains of CTCF and RUNX3 are highly conserved between human and mouse, sequence variability exists in their C‐terminal regions (Figure S6). Whether these divergences contribute to different L1 regulation remains to be investigated. Variations in the L1 5′ UTR region could also contribute. However, since mouse CTCF/RUNX3 failed to promote L1 transcription even in human cells (Figure 3I), this factor likely plays a minor role in the observed divergence. It is also noteworthy that while mouse cGAS inhibits L1 mRNA, mouse CTCF/RUNX3 overexpression has no significant effect (Figure 3A,J), suggesting the involvement of additional regulators in cGAS‐mediated L1 repression in mouse cells. These aspects remain unresolved and warrant further investigation.

### 3.4 cGAS‐Induced L1 mRNA Exacerbates Cellular Senescence in a MAVS‐Dependent Manner

**FIGURE 4:**
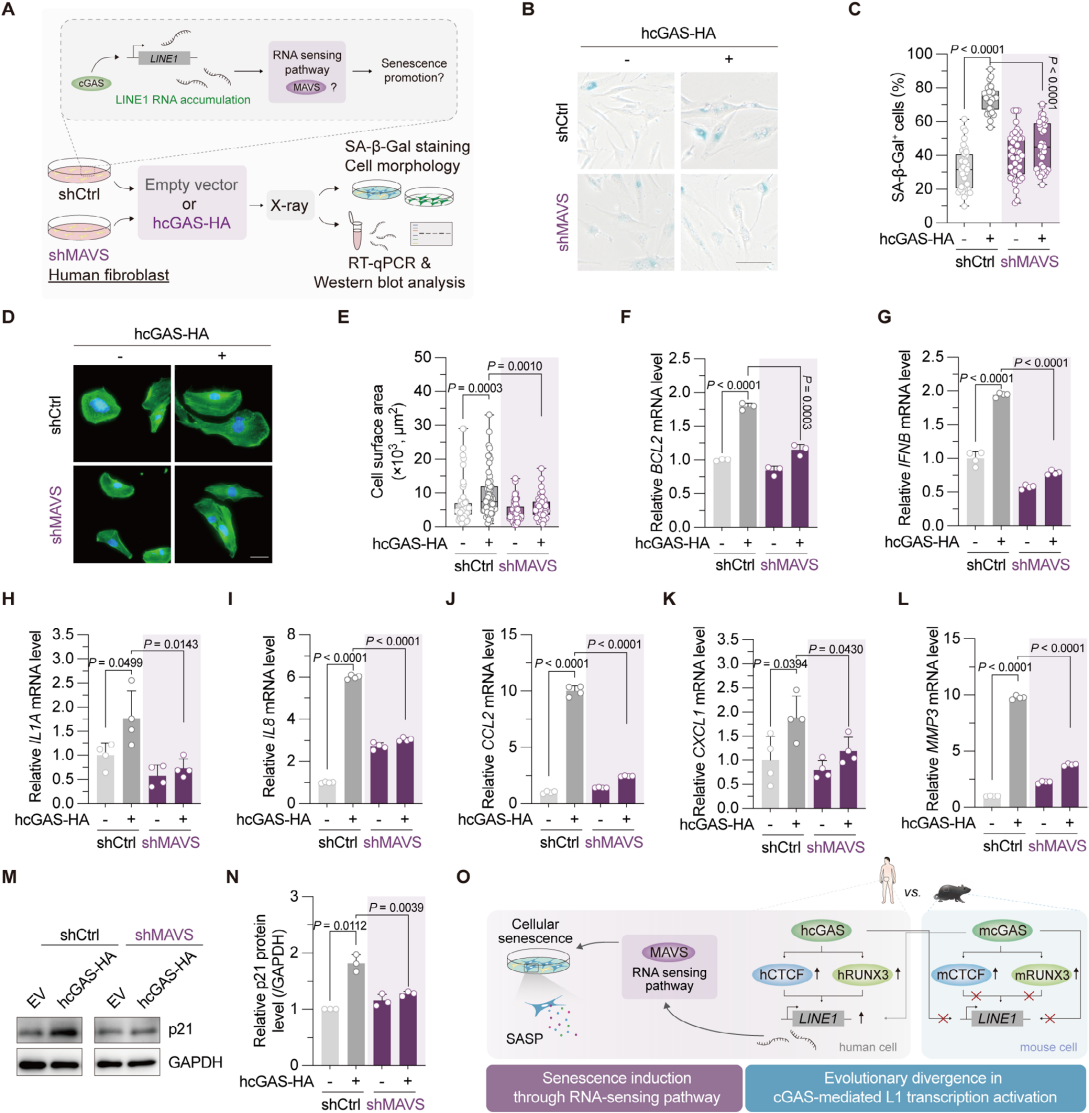
Human cGAS promotes cellular senescence through MAVS‐mediated RNA‐sensing pathway. (A) Illustration of the experimental design. HCA2‐hTERT cells, either with or without MAVS depletion, were transfected with a vector encoding human cGAS, followed by treatment with 6 Gy X‐irradiation. Cells were harvested on Day 6 postirradiation for senescence‐associated β‐galactosidase (SA‐β‐Gal) staining, cell size measurement, RT‐qPCR, and Western blot analysis. (B, C) SA‐β‐Gal staining in MAVS‐depleted HCA2‐hTERT cells, with or without transfection of human cGAS. Representative images are shown in (B) (scale bar: 50 μm), and the quantification results are shown in (C). (D, E) Analysis of cell surface area with phalloidin staining (scale bar: 50 μm). (F–L) The mRNA levels of antiapoptotic factor, *IFNB* and SASP factors in HCA2‐hTERT cells stably expressing the indicated shRNA, with or without human cGAS transfection. (M, N) The effect of human cGAS overexpression on p21 protein levels in HCA2‐hTERT cells with or without MAVS knockdown. The empty vector was transfected as the negative control for all the experiments. (O) Working model. Human cGAS facilitates L1 transcription by promoting the expression of CTCF and RUNX3. While mouse cGAS similarly upregulates these two factors, mouse CTCF and RUNX3 do not activate L1 transcription, revealing a species‐specific divergence in this downstream mechanism. Consequently, the human cGAS‐triggered increase in *L1* mRNA drives MAVS‐mediated induction of cellular senescence and expression of SASP factors.

Cytosolic RNA activates RNA‐sensing pathways, triggering the expression of interferons and inflammatory factors, many of which are SASP factors (Li, Zhu, et al. 2024). To assess whether cGAS‐mediated elevation of L1 mRNA similarly exacerbates the SASP and potentially cellular senescence, we depleted MAVS, a critical adaptor protein in the RNA‐sensing pathway, in HCA2‐hTERT cells (Figure 4A, Figure S7A). Consistent with the established role of cGAS in facilitating senescence, we found that human cGAS overexpression induced a remarkable increase in senescent cells (Yang et al. 2017), assayed by β‐Galactosidase staining. However, depletion of MAVS significantly abolished this effect in human cells (Figure 4B,C), indicating that cGAS‐triggered cellular senescence is partially dependent on MAVS. As expected, Mavs depletion did not affect cGAS‐overexpression‐induced cellular senescence in mouse cells (Figure S7B–D). Analysis of additional reported senescence markers, such as cell enlargement and upregulation of antiapoptotic factor BCL2 (Suryadevara et al. 2024), yielded consistent results (Figure 4D–F).

SASP factors comprise a panel of secretory interferons, cytokines, chemokines, and proteases. While cGAS has been identified as a key mediator of the SASP (Gluck et al. 2017; Victorelli et al. 2023; Yang et al. 2017), we sought to determine whether the RNA‐sensing pathway also contributes partially to this cGAS‐mediated SASP induction. To assess this, we compared the effect of cGAS overexpression on the mRNA levels of key SASP factors in control cells versus MAVS‐depleted cells. cGAS overexpression significantly increased the expression of *IFNB* and several critical SASP factors, including *IL1A*, *IL8*, *CCL2*, *CXCL1*, and *MMP3*. MAVS depletion significantly attenuated this induction in human cells but not in mouse cells (Figure 4G–L, Figure S7E–H). Western blot analysis revealed that cGAS overexpression also significantly upregulated the classical senescence marker p21. Again, this effect was abolished by MAVS depletion in human cells but not in mouse cells (Figure 4M,N, Figure S7I,J). Moreover, simultaneous depletion of both cGAS and MAVS did not further inhibit cellular senescence beyond single cGAS knockdown (Figure S8A–J), and L1 depletion abolished the pro‐senescence effect induced by cGAS overexpression (Figure S8K–Q). Overall, these data suggest that the L1–MAVS pathway mediates cGAS‐dependent senescence promotion.

Since DNA fragments generated by L1 reverse transcription also contribute to cellular senescence, we treated cells with the reverse transcriptase inhibitor 3TC. Although 3TC treatment significantly reduced senescence and inflammation as previously reported, cGAS overexpression still induced robust increases in the percentage of senescent cells and in p21 and SASP factor expression, even in the presence of 3TC (Figure S9A–G). Moreover, inhibition of the DNA sensing pathway using the STING inhibitor H‐151 attenuated senescence markers, while cGAS overexpression continued to promote cellular senescence under these conditions (Figure S9H–O). These data indicate that L1 promotes senescence through both DNA‐dependent and ‐independent mechanisms. To further confirm the role of L1 mRNA in activating RNA‐sensing pathways, we knocked down L1 in control and MAVS‐depleted cells. Our data showed that L1 depletion significantly attenuated IFNβ transcriptional activation and cellular senescence in control cells, but had minimal effect on these phenotypes in MAVS‐depleted cells (Figure S10).

Collectively, these data demonstrated that cGAS potentiates human cellular senescence, an effect that partially requires the L1‐mediated activation of the RNA‐sensing pathway (Figure 4O).

## 4 Discussion

Accumulating evidence demonstrates crucial roles for both cGAS and L1 retrotransposons in regulating cellular senescence and aging (De Cecco et al. 2019; Dou et al. 2017; Gluck et al. 2017; Simon et al. 2019; Yang et al. 2017). Intriguingly, complex interactions exist between cGAS and L1. L1 ORF2p induces DNA double‐strand breaks that activate DNA damage responses to drive senescence. Moreover, retrotransposition generates cytosolic DNA fragments that activate cGAS‐mediated innate immune responses and inflammatory cytokine production, promoting senescence (De Cecco et al. 2019; Simon et al. 2019). Our previous study demonstrated that cGAS restricts L1 expression by inducing ORF2p degradation (Zhen et al. 2023). Interestingly, cGAS enriches at L1 DNA repeats (Gentili et al. 2019). However, whether and how cGAS influences L1 transcription remained unclear. Here, we demonstrate that human cGAS promotes L1 transcription independently of its canonical enzymatic activity, suggesting cGAS may oppositely regulate L1 at transcriptional and posttranslational stages.

L1 mRNA upregulation occurs during early embryonic development and the aging process (De Cecco et al. 2019; Guo et al. 2014; Simon et al. 2019). Interestingly, L1 expression is essential for early embryonic development (Guo et al. 2014; Ma et al. 2025; Zhang et al. 2025). Despite elevated L1 mRNA levels, the rate of L1 retrotransposition—a process often considered mutagenic and harmful—remains low during early embryonic development and in germ cells (Kano et al. 2009; Newkirk et al. 2017; Richardson et al. 2017). This uncoupling of transcription and retrotransposition preserves the beneficial and essential role of L1 mRNA in development while minimizing its potential to destabilize the genome. The precise balance between these closely linked processes confers a fitness advantage and might be strongly favored by selection. The mechanisms underlying this regulation, however, remain poorly understood. Our finding that human cGAS promotes L1 transcription, combined with its known role in suppressing retrotransposition (Zhen et al. 2023), provides a potential mechanism for this regulation. Paradoxically, cGAS‐mediated L1 transcription may potentiate cellular senescence as age‐associated derepression of L1 loci progresses, ultimately promoting biological aging. This “double‐edged sword” function of cGAS, which is beneficial in early life but detrimental later, aligns with the Antagonistic Pleiotropy theory (Williams 1957), which posits that traits favored by selection for early‐life benefits can become harmful in post‐reproductive ages as selective pressure diminishes, thereby explaining the evolutionary origin of aging.

Mechanistically, human cGAS facilitates L1 transcription by upregulating the L1 regulators CTCF and RUNX3, whereas mouse cGAS does not promote L1 transcription (Figure 4O). Further mechanistic analysis revealed that although mouse cGAS retains the ability to upregulate CTCF and RUNX3 in mouse cells, these mouse homologs fail to elevate L1 mRNA levels. Do sequence differences between human and mouse drive this divergence? Why did this regulatory link evolve in humans? Is it present in other mammalian clades? And what are the biological consequences of this evolutionary event? These fascinating questions require further investigation. Intriguingly, analysis of publicly accessible data (GSE284706) revealed that cGAS depletion in mice significantly altered the expression of certain long terminal repeat (LTR) sequences (Figure S11). Elucidating the regulatory mechanisms of this alteration and investigating its evolutionary conservation are key future research directions. Additionally, leveraging methods of comparative biology, our recent work indicated that the role of cGAS in regulating homologous recombination repair is significantly altered in the longest‐lived rodent, the naked mole‐rat (Chen et al. 2025, 2026; Tang et al. 2025; Wang et al. 2025). How cGAS in long‐lived animals regulates L1 transcription and retrotransposition is also an intriguing question to be explored.

Although diverse mechanisms, including DNA methylation, histone modifications, transcriptional regulators, and posttranscriptional processes, govern L1 expression (Li and Liu 2025), our study specifically examines the roles of RUNX3 and CTCF in cGAS‐mediated transcriptional activation. Potential contributions from yet‐to‐be‐identified factors warrant further investigation.

Chronic inflammation is both a hallmark and driver of aging (Kroemer et al. 2025). Earlier studies identified multiple factors that sense microbial motifs to initiate innate immune responses. Recent findings demonstrate that these same factors can also be activated by nonmicrobial signals, including endogenous DNA and RNA (Chen and Nunez 2010), triggering sterile inflammation. Among these factors, MAVS plays a critical role in mediating cellular antiviral signaling triggered by RNA, whereas cGAS serves as a primary DNA sensor. Furthermore, a growing body of literature links cGAS to diverse biological events, many independent of its canonical enzymatic function. Our study reveals that cGAS, independent of its canonical function, promotes L1 transcription, increasing cytosolic L1 mRNA, which may then activate MAVS‐dependent pathways, contributing to sterile inflammation. Supporting our findings, a previous study demonstrated that LINE1 activates interferon production through RNA‐sensing pathways in the context of autoimmunity (Zhao et al. 2018). In summary, our research establishes a novel crosstalk between the factors within the nucleotide‐sensing network. Moreover, we highlight their interaction in the regulation of cellular senescence, suggesting potential targets for interventions against aging.

## Author Contributions

Y.C. and Z.M.: conceptualization and supervision; Y.C., Z.M. and Y.J.: funding acquisition; Z.C., L.C., X.C., and Z.Z.: experimentation, data acquisition and analysis; H.W. and H.T.: bioinformatics analysis; Y.C. and Z.C.: original draft writing; all authors are involved in manuscript review and editing.

## Funding

This work was supported by National Natural Science Foundation of China (82522035, 82225017), National Key Research and Development Program of China (2022YFA1103700), National Natural Science Foundation of China (32470796, 32200595, 32421002, 82361148131, 32270750, 32171288, 32471341), Fundamental Research Funds for the Central Universities (22120240273, 22120250374), and Peak Disciplines (Type IV) of Institutions of Higher Learning in Shanghai.

## Conflicts of Interest

The authors declare no conflicts of interest.

## Supporting information


**Figure S1:** The effect of human cGAS overexpression on *L1* mRNA level.
**Figure S2:** ChIP‐seq analysis of cGAS binding patterns on L1ME3A and L1MB3 subfamilies.
**Figure S3:** Correlation analysis between *CGAS* expression levels and *CTCF* or *RUNX3* expression levels.
**Figure S4:** Molecular mechanisms underlying *L1* transcription regulation.
**Figure S5:** Cross‐species analysis of the impact of CTCF and RUNX3 on *L1* mRNA levels.
**Figure S6:** Conservation analysis of human and mouse RUNX3 and CTCF protein.
**Figure S7:** The impact of mouse cGAS on senescence in Mavs‐depleted mouse cells.
**Figure S8:** Regulatory mechanism of cGAS‐triggered cellular senescence.
**Figure S9:** L1 RNA triggers cellular senescence.
**Figure S10:** L1‐MAVS axis promotes cellular senescence in human cells.
**Figure S11:** The effect of cGAS depletion on the expression levels of repetitive elements beyond L1.
**Table S1:** shRNA and sgRNA sequences used in this study.
**Table S2:** qPCR primers used in this study.

## Data Availability

All data generated during this study are included in this published article and its Supporting Information files.
