# Supplementary material for: Human cGAS Drives LINE‐1 Transcriptional Activation to Trigger MAVS‐Dependent Cellular Senescence
Source: Aging Cell. 2026 Apr 9;25(4):e70484. doi: 10.1111/acel.70484 (PMC13065494; doi:10.1111/acel.70484)
Supplement: Supplementary file 1 — Figure S1: The effect of human cGAS overexpression on L1 mRNA level. Figure S2: ChIP‐seq analysis of cGAS binding patterns on L1ME3A and L1MB3 subfamilies. Figure S3: Correlation analysis between CGAS expression levels and CTCF or RUNX3 expression levels. Figure S4: Molecular mechanisms underlying L1 transcription regulation. Figure S5: Cross‐species analysis of the impact of CTCF and RUNX3 on L1 mRNA levels. Figure S6: Conservation analysis of human and mouse RUNX3 and CTCF protein. Figure S7: The impact of mouse cGAS on senescence in Mavs‐depleted mouse cells. Figure S8: Regulatory mechanism of cGAS‐triggered cellular senescence. Figure S9: L1 RNA triggers cellular senescence. Figure S10: L1‐MAVS axis promotes cellular senescence in human cells. Figure S11: The effect of cGAS depletion on the expression levels of repetitive elements beyond L1. Table S1: shRNA and sgRNA sequences used in this study. Table S2: qPCR primers used in this study. [file ACEL-25-e70484-s001.docx]

**Supporting Information**

**Human cGAS Drives LINE-1 Transcriptional Activation to Trigger MAVS-Dependent Cellular Senescence**

Zhixi Chen^#^, Lingjiang Chen^#^, Xinyu Chen, Hao Wang, Huanyin Tang, Zhengyi Zhen, Ying Jiang, Zhiyong Mao^*^, Yu Chen^*^

Shanghai Key Laboratory of Maternal Fetal Medicine, Clinical and Translational Research Center of Shanghai First Maternity and Infant Hospital, Frontier Science Center for Stem Cell Research, School of Life Sciences and Technology, Tongji University, Shanghai 200092, China

^#^ Zhixi Chen and Lingjiang Chen contributed equally to this work.

* Corresponding author: Yu Chen & Zhiyong Mao. Email: y_chen@tongji.edu.cn; zhiyong_mao@tongji.edu.cn.

**
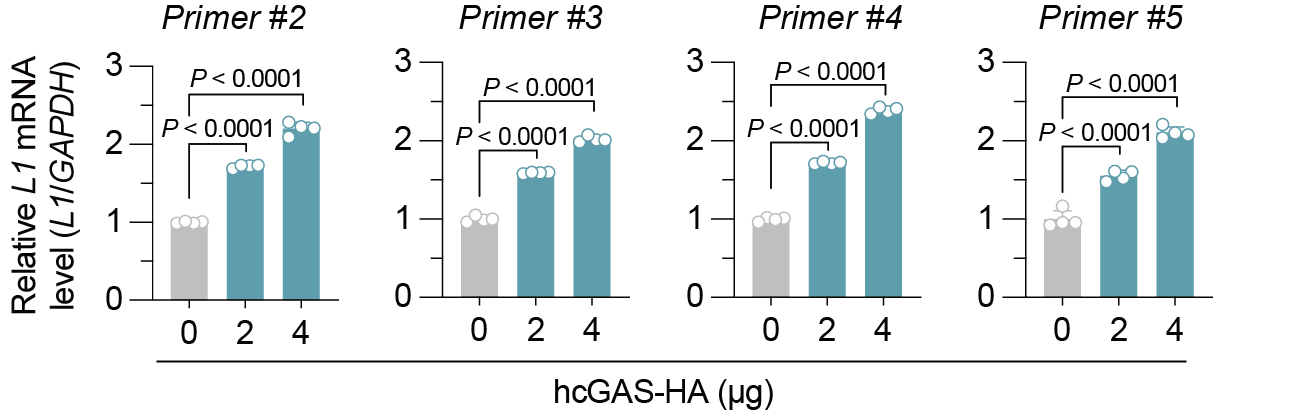
**

**Figure S1. The effect of human cGAS overexpression on *L1* mRNA level**

Analysis of *L1* mRNA levels in HeLa cells following overexpression of indicated amounts of human cGAS. Data are presented as mean values ± s.d.. Statistical significance was determined using t-test.


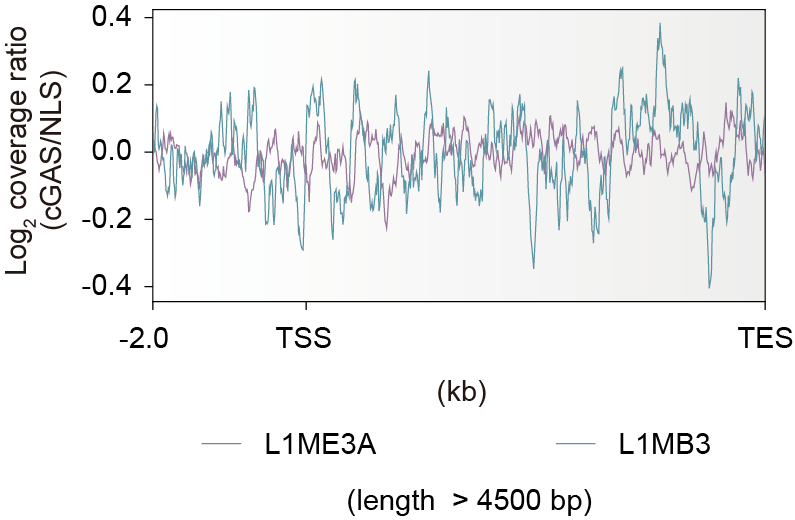


**Figure S2. ChIP-seq analysis of cGAS binding patterns on L1ME3A and L1MB3 subfamilies**

L1ME3A and L1MB3 elements with length > 4500 bp were used for analysis. TSS, transcription start site; TES, transcription end site.


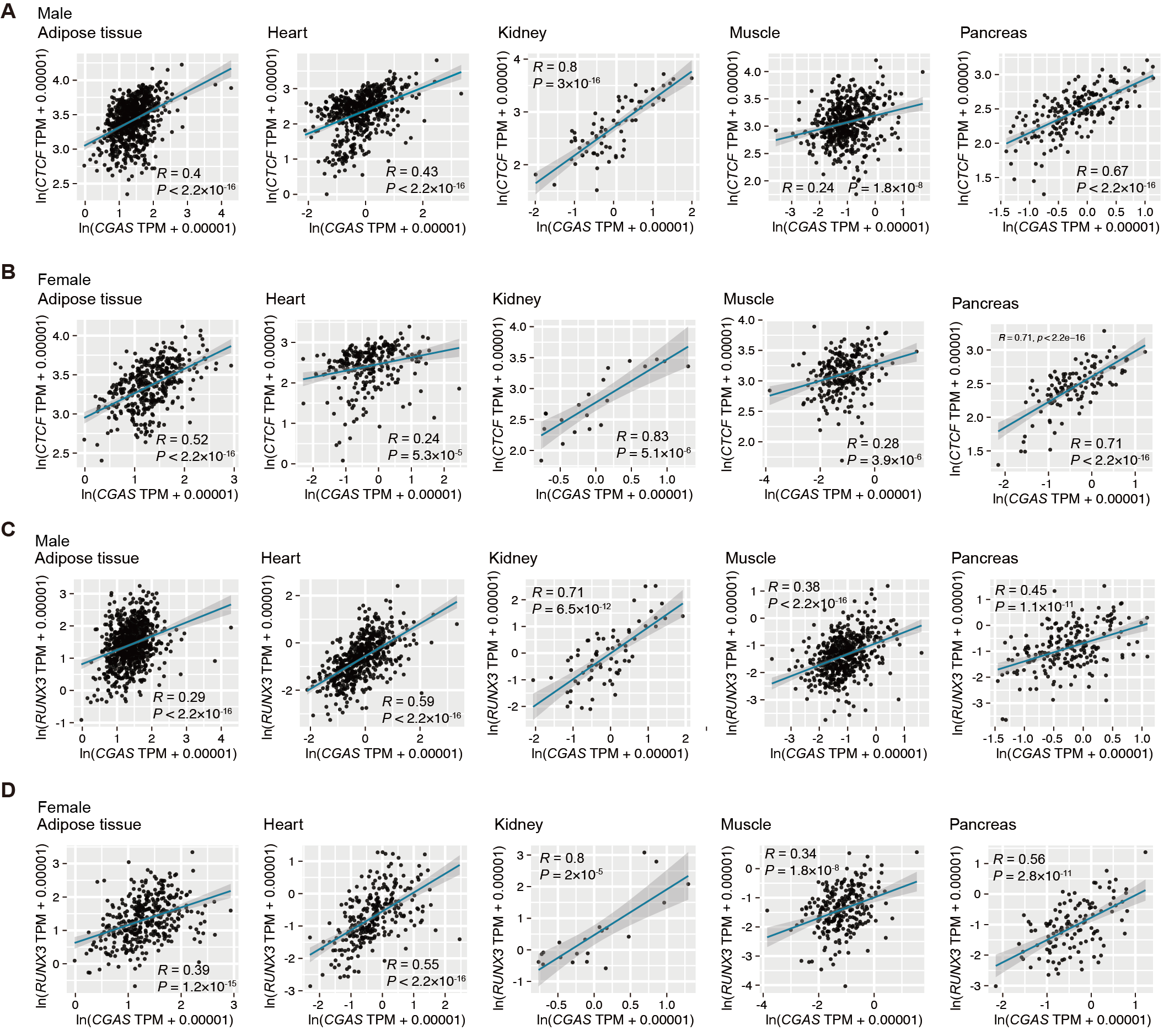


**Figure S3. Correlation analysis between *CGAS* expression levels and *CTCF* or *RUNX3* expression levels**

**A**-**B**. Correlation analysis of mRNA levels of *CGAS* and *CTCF* across indicated human tissues in males (A) and females (B). **C**-**D**. Correlation analysis of mRNA levels of *CGAS* and *RUNX3* across indicated human tissues in males (C) and females (D).


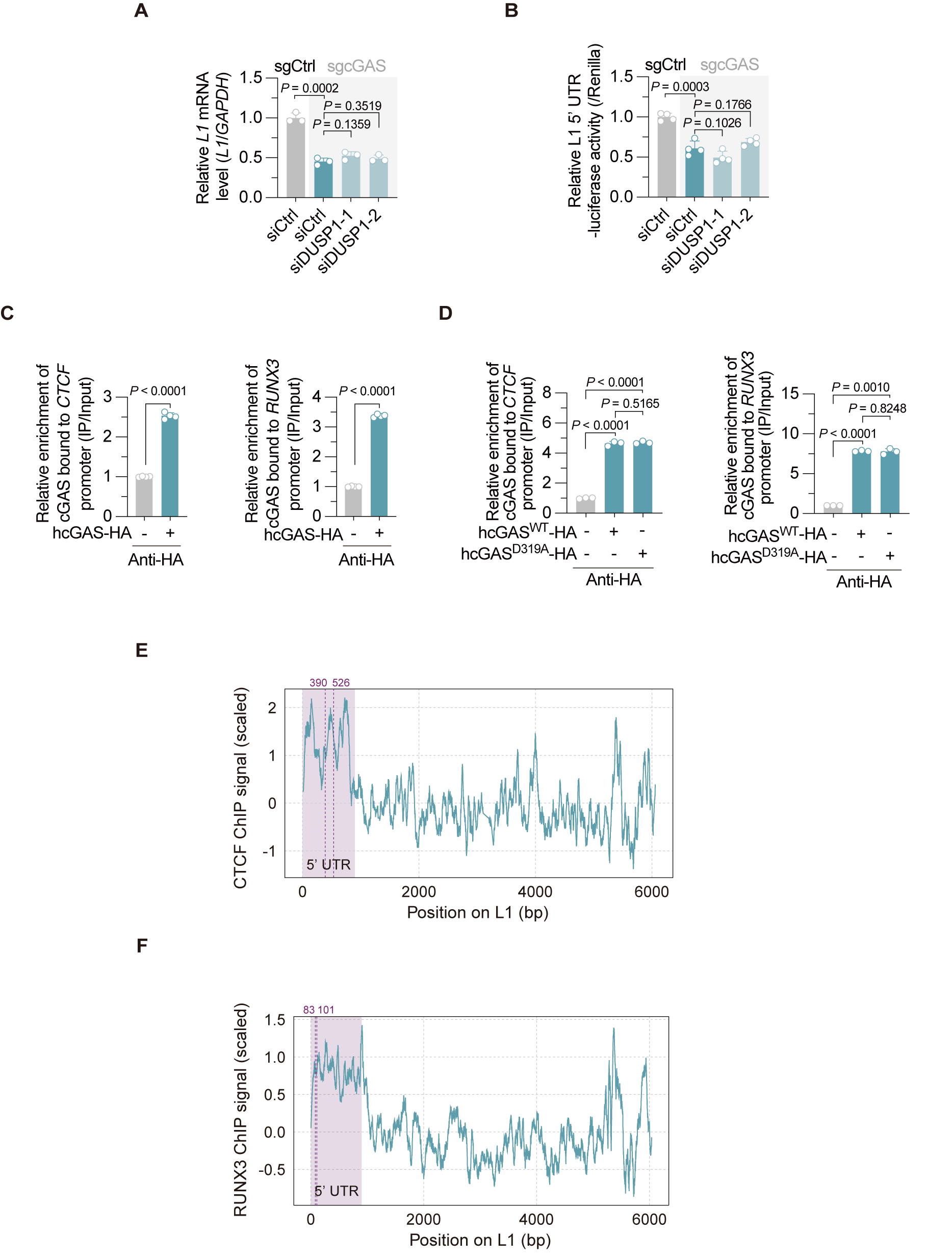


**Figure S4. Molecular mechanisms underlying *L1* transcription regulation**

**A**. Analysis of *L1* mRNA levels in cGAS-depleted HeLa cells with or without DUSP1 knockdown. **B**. The effect of DUSP1 knockdown on L1 5’ UTR promoter activity in wild-type and cGAS-depleted HeLa cells. **C**. cGAS enrichment at *CTCF* and *RUNX3* promoters analyzed by ChIP-qPCR. Cells transfected with empty vectors served as the negative control. **D**. Analysis of the enrichment of cGAS and its enzymatic mutant at *CTCF* or *RUNX3* promoter by ChIP-qPCR. The empty vector was transfected as the negative control. Data are presented as mean values ± s.d.. **E**-**F**. Both CTCF and RUNX3 are enriched in the 5’ UTR of the human-specific L1HS sequence. Publicly accessible ChIP-seq data (GSM749695 and GSM1010893) were used. Previously reported RUNX3 (+83 to +101) and CTCF (+390 to +526) binding sites were annotated.

**
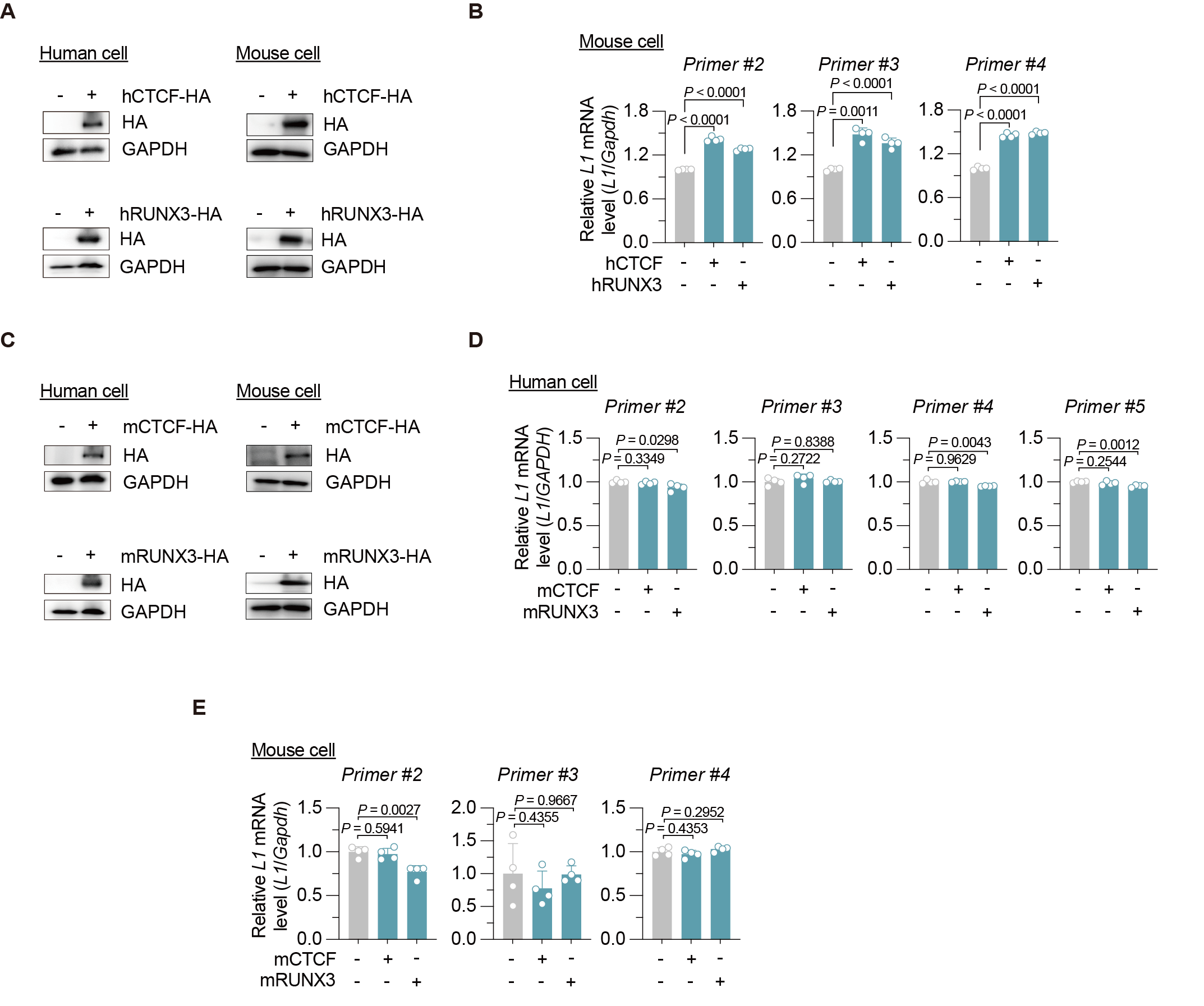
**

**Figure S5. Cross-species analysis of the impact of CTCF and RUNX3 on *L1* mRNA levels**

**A**. Validation of human CTCF and RUNX3 protein expression by Western blot. **B**. The effect of overexpressing human CTCF or RUNX3 on *L1* mRNA levels in MSFs. **C**. Validation of mouse CTCF and RUNX3 protein expression by Western blot. **D**. The effect of overexpressing mouse CTCF or RUNX3 on *L1* mRNA level in human HCA2-hTERT cells. **E**. The effect of overexpressing mouse CTCF or RUNX3 on *L1* mRNA level in MSFs. Data are presented as mean values ± s.d.. The empty vector was transfected as the negative control for all the experiments.

**
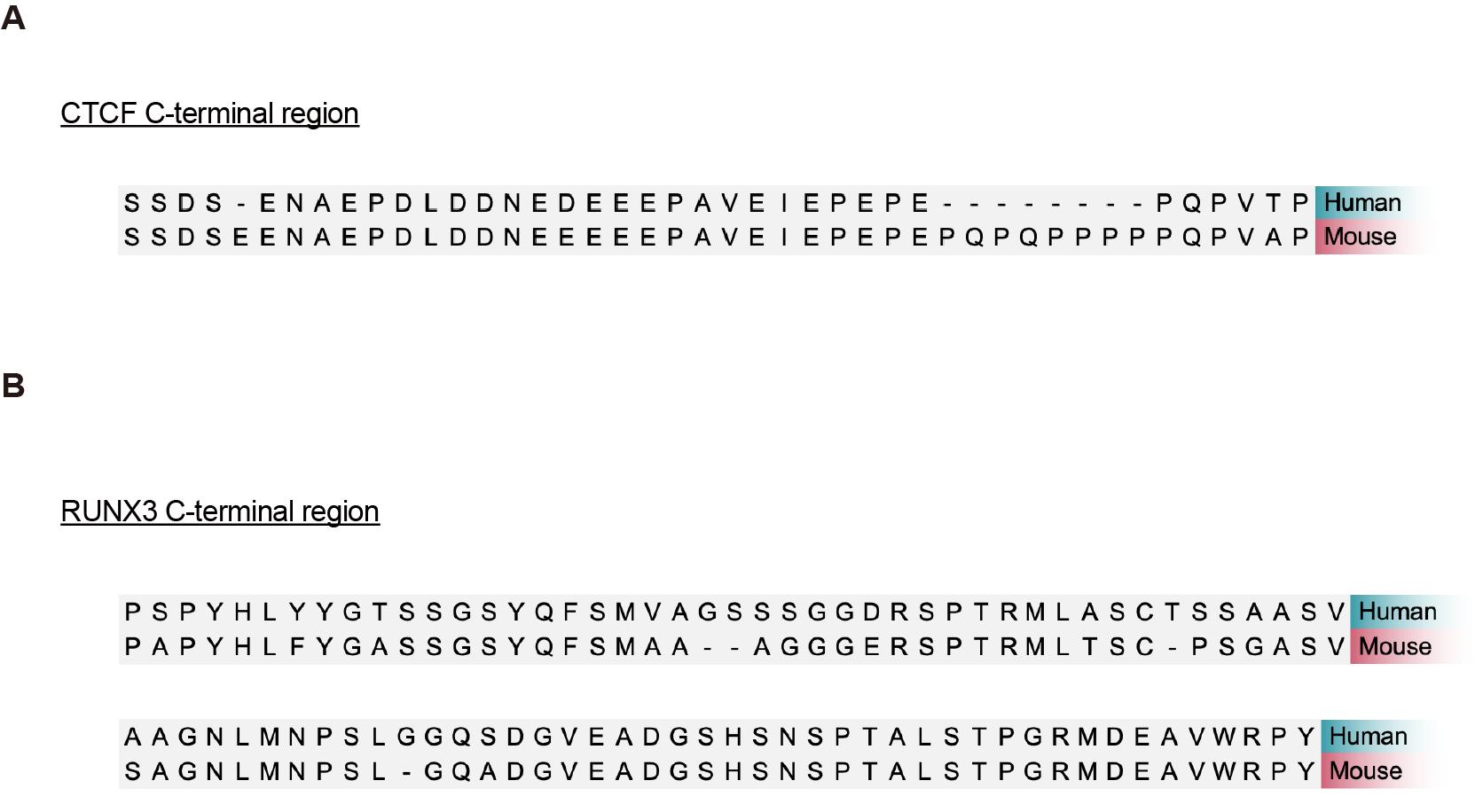
**

**Figure S6. Conservation analysis of human and mouse RUNX3 and CTCF protein**

Alignment of the C-terminal domains of human and mouse RUNX3 and CTCF protein sequences using MEGAX.

**
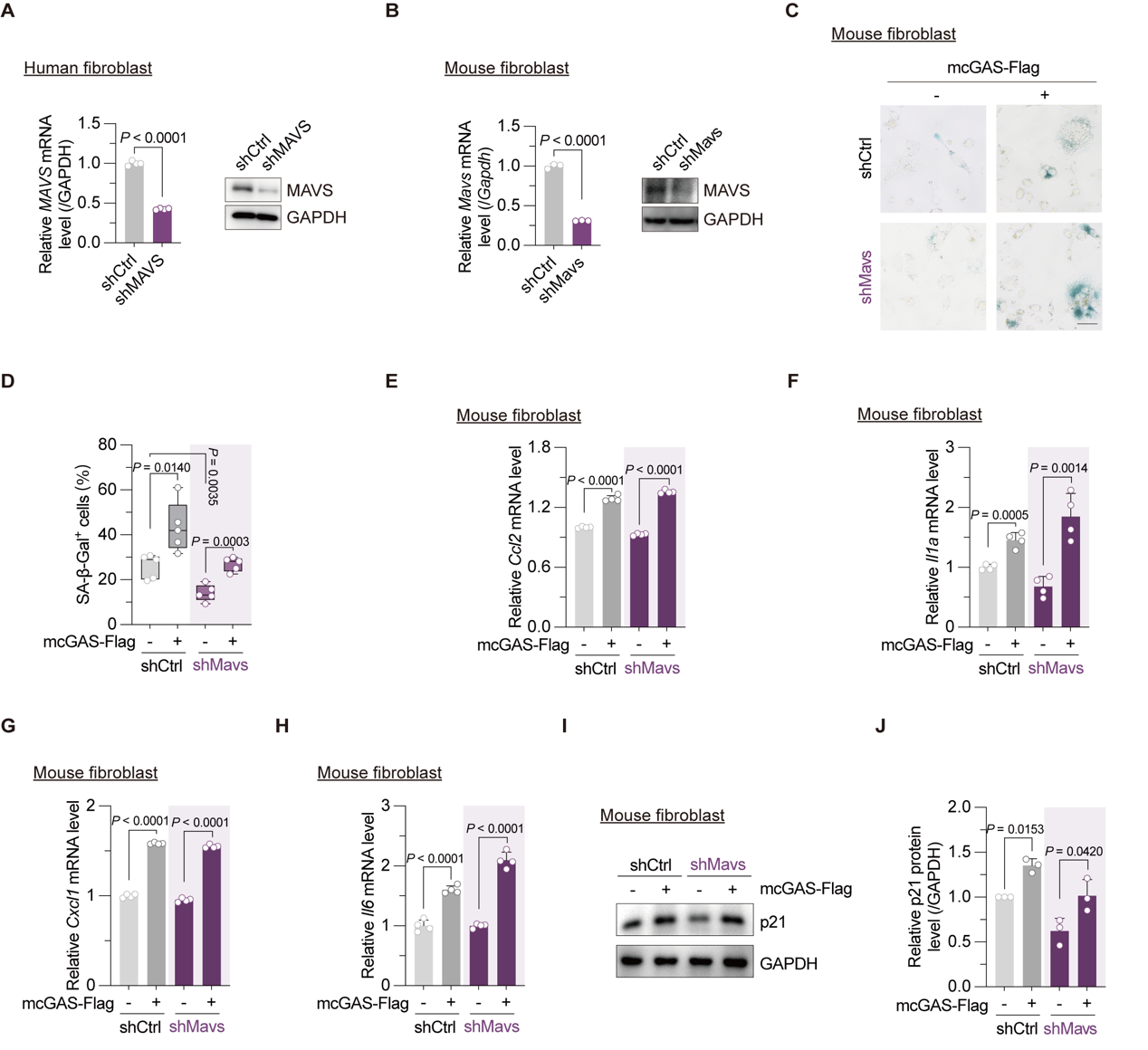
**

**Figure S7.** **The impact of mouse cGAS on senescence in Mavs-depleted mouse cells**

**A**-**B**. RT-qPCR and Western blotting analysis of the MAVS knockdown efficiency in HCA2-hTERT cells and mouse fibroblasts. **C**-**D**. SA-β-Gal staining in irradiated mouse fibroblasts. Control or Mavs-depleted mouse fibroblasts were transfected with or without mouse cGAS (scale bar: 50 µm). **E**-**H**. mRNA levels of SASP factors in irradiated mouse fibroblasts transfected with cGAS, with or without Mavs depletion. **I**-**J**. The effect of mouse cGAS overexpression on p21 protein levels in mouse fibroblasts with or without Mavs depletion. Data are presented as mean values ± s.d.. The empty vector was transfected as the negative control for all the experiments.


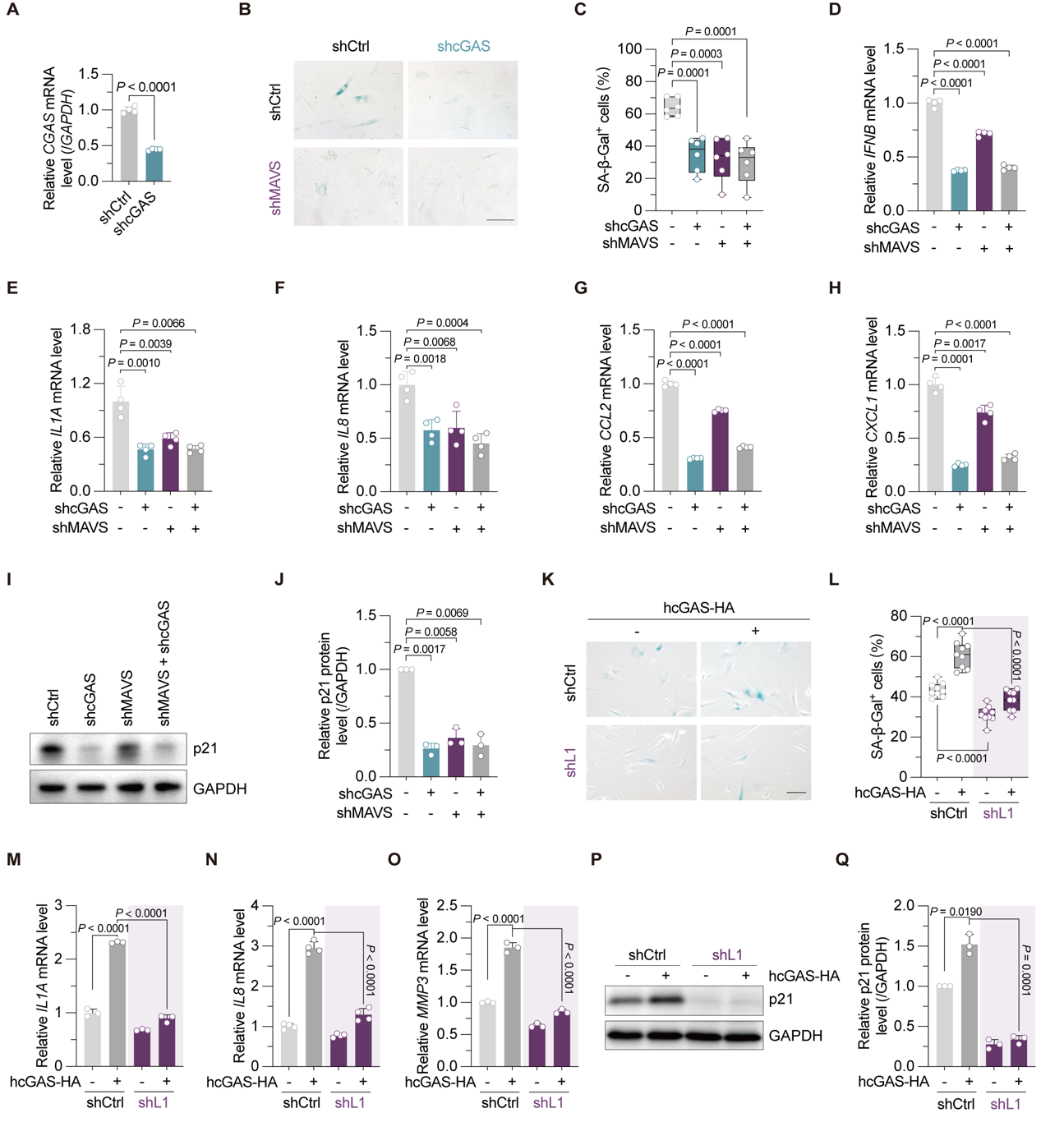


**Figure S8. Regulatory mechanism of cGAS-triggered cellular senescence**

**A**. RT-qPCR analysis of the cGAS knockdown efficiency in HCA2-hTERT cells. **B**-**C**. SA-β-Gal staining in irradiated HCA2-hTERT cells with cGAS or/and MAVS depletion (scale bar: 100 µm). **D**-**H**. mRNA levels of SASP factors in irradiated HCA2-hTERT cells with cGAS or/and MAVS depletion. **I**-**J**. Western blotting analysis on p21 protein levels. **K**-**L**. SA-β-Gal staining in irradiated HCA2-hTERT cells with L1 depletion and cGAS overexpression (scale bar: 100 µm). **M**-**O**. mRNA levels of SASP factors in irradiated HCA2-hTERT cells with cGAS overexpression or/and L1 depletion. **P**-**Q**. Western blotting analysis on p21 protein levels. Data are presented as mean values ± s.d..


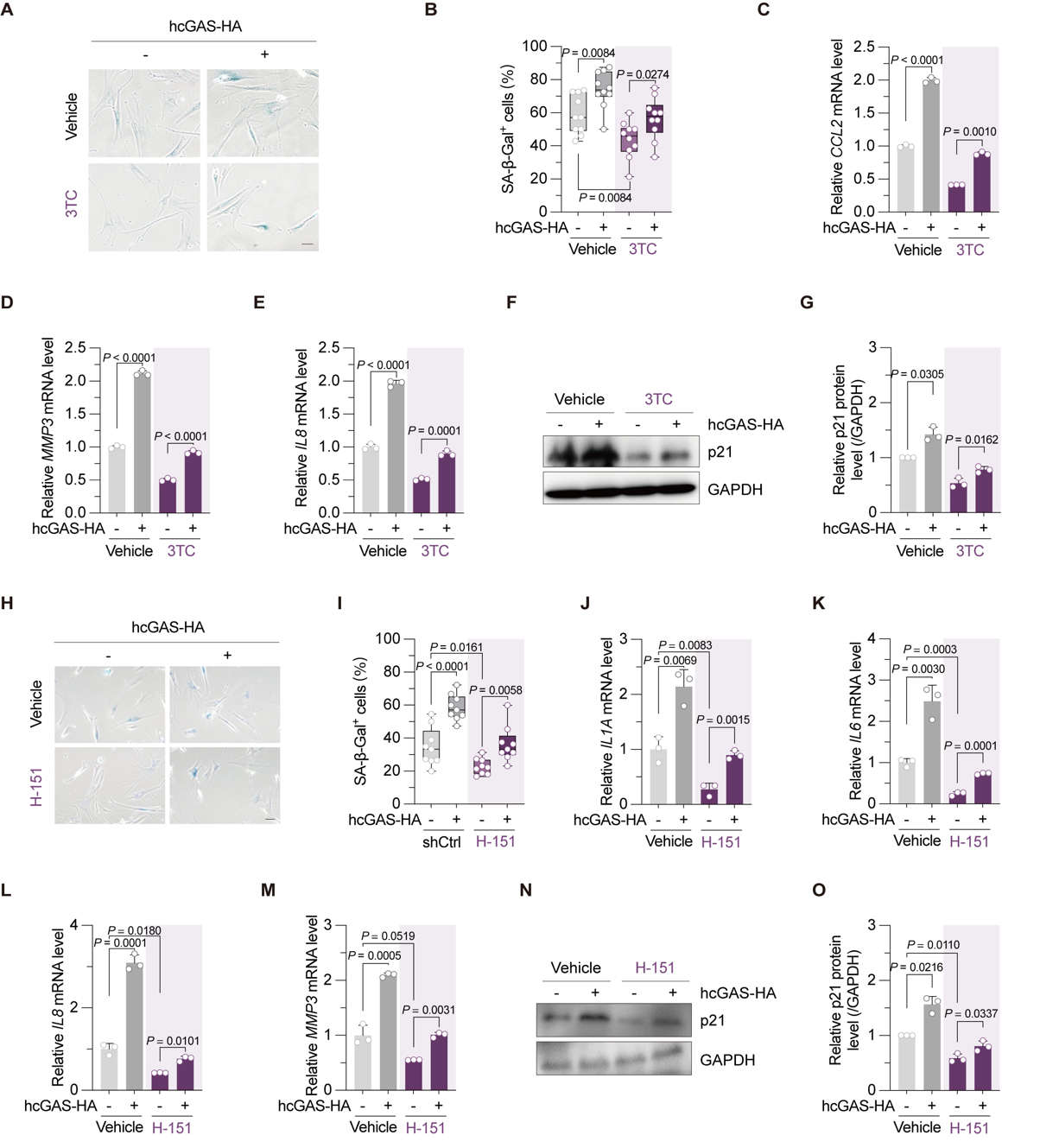


**Figure S9. L1 RNA triggers cellular senescence**

**A**-**B**. SA-β-Gal staining in irradiated human cGAS transfected HCA2-hTERT cells, with or without 3TC treatment (scale bar: 50 µm). **C**-**E**. The mRNA levels of SASP factors in irradiated human cGAS transfected HCA2-hTERT cells, with or without 3TC treatment. **F**-**G**. The effect of human cGAS overexpression on p21 protein levels. **H**-**I**. SA-β-Gal staining in irradiated human cGAS transfected HCA2-hTERT cells, with or without H-151 treatment (scale bar: 50 µm). **J**-**M**. The mRNA levels of SASP factors in irradiated human cGAS transfected HCA2-hTERT cells, with or without H-151 treatment. **N**-**O**. The effect of human cGAS overexpression on p21 protein levels. The empty vector was transfected as the negative control for all the experiments.


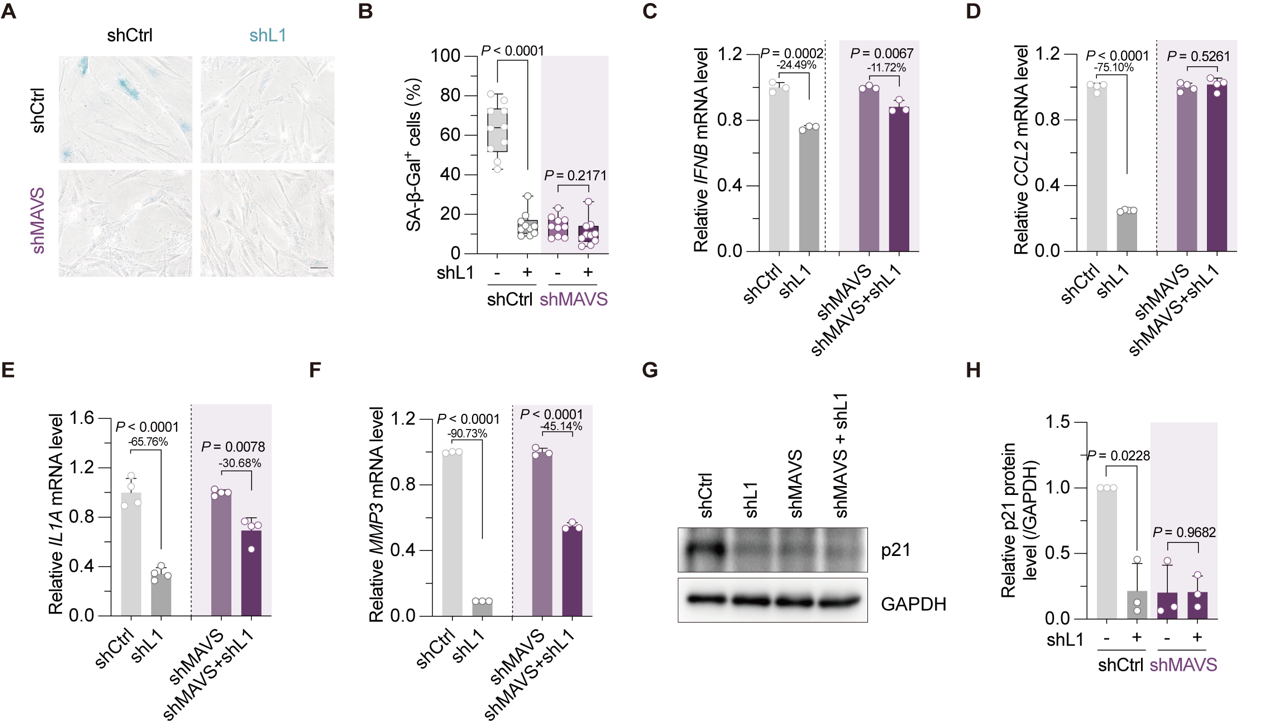


**Figure S10. L1-MAVS axis promotes cellular senescence in human cells**

**A**-**B**. SA-β-Gal staining in irradiated HCA2-hTERT cells with L1 and/or MAVS depletion (scale bar: 50 µm). **C**-**F**. The mRNA levels of SASP factors in irradiated HCA2-hTERT cells with L1 and/or MAVS depletion. **G**-**H**. Analysis of p21 expression in irradiated HCA2-hTERT cells with L1 and/or MAVS depletion. Data are presented as mean values ± s.d..


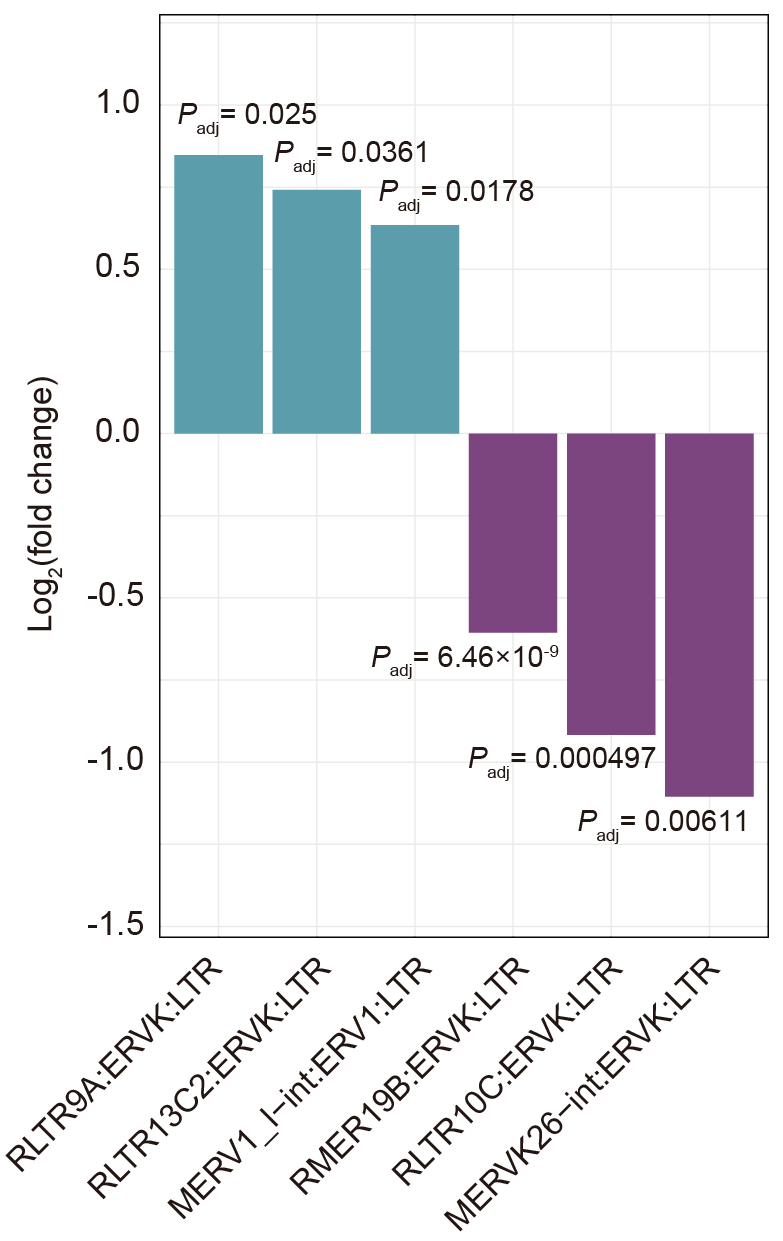


**Figure S11. The effect of cGAS depletion on the expression levels of repetitive elements beyond L1**

A published RNA-seq dataset (GSE284706) revealed that cGAS depletion in mice significantly altered the expression of certain long terminal repeat (LTR) sequences.

**Table S1. shRNA and sgRNA sequences used in this study**

| **Target** | **Sequence** |
| --- | --- |
| CTCF (human) shRNA | CAAGAATGAGAAGCGCTTTAA |
| RUNX3 (human) shRNA | ACCACCTCTACTACGGGACAT |
| MAVS (human) shRNA | ATGTGGATGTTGTAGAGATTC |
| Mavs (mouse) shRNA | CCAGTGCTGATCTATTAGGAA |
| L1 (human) shRNA | AAGACACATGCACACGTATGT |
| cGAS (human) shRNA-1 | CGTGAAGATTTCTGCACCTAA |
| cGAS (human) shRNA-2 | GGCTATCCTTCTCTCACAT |
| cGAS (human) sgRNA-1 | GGCCGCCCGTCCGCGCAACT |
| cGAS (human) sgRNA-2 | ACACTCGTGCATATTACTTT |

**Table S2. qPCR primers used in this study**

| **Target** | **Sequence** |
| --- | --- |
| *L1*-1 (human) | CAAACACCGCATATTCTCACTCA  CTTCCTGTGTCCATGTGATCTCA |
|  |  |
| *L1*-2 (human) | GCCAAGATGGCCGAATAGGA |
|  | AAATCACCCGTCTTCTGCGT |
| *L1*-3 (human) | CGAGATCAAACTGCAAGGCG  CCGGCCGCTTTGTTTACCTA |
| *L1*-4 (human) | TAAACAAAGCGGCCGGGAA |
|  | AGAGGTGGAGCCTACAGAGG |
| *L1*-5 (human) | AGAGAGCAGTGGTTCTCCCA |
|  | CAGTCTGCCCGTTCTCAGAT |
| *L1*-1 (mouse) | CTGCCTTGCAAGAAGAGAGC |
|  | AGTGCTGCGTTCTGATGATG |
| *L1*-2 (mouse) | ATCTGTCTCCCAGGTCTGCT |
|  | TCCTCCGTTTACCTTTCGCC |
| *L1*-3 (mouse) | GCTTCGGTGAAGTAGCTGGA |
|  | TTCGTTAGAGTCACGCCGAG |
| *L1*-4 (mouse) | AGCCAAATGGATGGACCTGG |
|  | AAGGAGGGGCATAGTGTCCA |
| *CTCF* (human) | AGACTTACCAGAGACGCCG |
|  | TCCAGCTGTTCCATCATCACC |
| *Ctcf* (mouse) | AGACTTACCAGAGACGCCG |
|  | CCAGCTGTTCCATCATTACC |
| *CTCF* (ChIP assay) | CCAACCCTGTACGGGTTCAG  GGGCGCCAACTTTCAAAAGC |
| *RUNX3* (human) | GAACTACTCCGCTGAGCTGC |
|  | GAACACAGTGATGGTCAGGG |
| *Runx3* (mouse) | GAACTACTCCGCCGAGCTGC |
|  | GAACACGGTGATTGTGAGCG |
| *RUNX3* (ChIP assay) | AAAATCCCGTGTGGGGTGGA TCGTAGCTCCCTGACGCTG |
| *IFNB* (human) | ATGACCAACAAGTGTCTCCTCC |
|  | GGAATCCAAGCAAGTTGTAGCTC |
| *CCL2* (human) | AGTCTCTGCCGCCCTTCT |
|  | GTGACTGGGGCATTGATTG |
|  |  |
| *MMP3* (human) | CCCACCTTACATACAGGATTGTGA |
|  | CCCAGACTTTCAGAGCTTTCTCA |
| *IL8* (human) | AGACAGCAGAGCACACAAGC |
|  | ATGGTTCCTTCCGGTGGT |
| *CXCL1* (human) | TCCTGCATCCCCCATAGTTA |
|  | CTTCAGGAACAGCCACCAGT |
| *IL1A* (human) | GGTTGAGTTTAAGCCAATCCA |
|  | TGCTGACCTAGGCTTGATGA |
| *BCL2* (human) | GGTGGGGTCATGTGTGTGG |
|  | CGGTTCAGGTACTCAGTCATCC |
| *Ifnb* (mouse) | CAGCTCCAAGAAAGGACGAAC |
|  | GGCAGTGTAACTCTTCTGCAT |
| *Ccl2* (mouse) | TTAAAAACCTGGATCGGAACCAA |
|  | GCATTAGCTTCAGATTTACGGGT |
| *Mmp3* (mouse) | ACATGGAGACTTTGTCCCTTTTG |
|  | TTGGCTGAGTGGTAGAGTCCC |
| *Il1a* (mouse) | CGAAGACTACAGTTCTGCCATT |
|  | GACGTTTCAGAGGTTCTCAGAG |
|  |  |
| *Cxcl1* (mouse) | CTGGGATTCACCTCAAGAACATC |
|  | CAGGGTCAAGGCAAGCCTC |
| *MAVS* (human) | AGAGAATTCAGAGCAAGCCCT |
|  | ACCTGCTGTGTGGGTACT |
| *Mavs* (mouse) | CTGCCTCACAGCTAGTGACC |
|  | CCGGCGCTGGAGATTATTG |
| *GAPDH* | ATGACATCAAGAAGGTGGTG |
|  | CATACCAGGAAATGAGCTTG |
